# Assessing time processing ability and daily time management in persons with dementia: Psychometric properties of three instruments

**DOI:** 10.1111/1440-1630.12827

**Published:** 2022-06-29

**Authors:** Ann‐Christine Persson, Marika C. Möller, Lena Dahlberg, Monika Löfgren, Gunnel Janeslätt

**Affiliations:** ^1^ Department of Clinical Sciences, Danderyd Hospital Karolinska Institutet Stockholm Sweden; ^2^ Department of Rehabilitation Medicine Danderyd University Hospital Stockholm Sweden; ^3^ School of Health and Welfare Dalarna University Falun Sweden; ^4^ Aging Research Center Karolinska Institutet & Stockholm University Solna Sweden; ^5^ Department of Public Health and Caring Sciences Disability and Habilitation, Uppsala University Uppsala Sweden; ^6^ Center for Clinical Research in Dalarna Falun Sweden

**Keywords:** Alzheimer's disease, cognition, measurement, time orientation, time perception

## Abstract

**Introduction:**

Persons with dementia experience time‐related problems, but there is a lack of instruments evaluating their time processing ability and daily time management. This study aimed to evaluate the psychometric properties of the instruments KaTid®‐Senior measuring time processing ability, and Time‐S© Senior and Time‐Proxy© measuring daily time management for persons with dementia.

**Methods:**

Persons with dementia (*n* = 53) and their significant others (*n* = 49) participated in the study. Rasch analyses were used to evaluate the instruments' rating scale functioning; internal scale validity; person‐response validity; unidimensionality; person‐separation reliability; and internal consistency. Versions excluding items with poor fit to the Rasch model were also evaluated.

**Results:**

Overall, the Rasch analyses showed acceptable psychometric properties. All instruments met the criteria for unidimensionality and the reliability was good. More challenging items should be added in KaTid‐Senior for better targeting of persons with mild dementia. Person‐response validity issues in Time‐S Senior need to be addressed.

**Conclusion:**

The instruments can validly and reliably be used to assess time processing ability and daily time management in persons with dementia in clinical research and healthcare settings. In turn, this can contribute to the development of methods to compensate for impaired time processing ability and daily time management. The assessments can also increase the possibility of early detection of impaired time processing ability and daily time management, thereby facilitating adequate timing of interventions and enhanced occupational performance.

**Key Points for Occupational Therapy**

1. KaTid®‐Senior can validly and reliably assess time processing ability in dementia.
2. Time‐S© Senior and Time‐Proxy© can validly and reliably assess daily time management in dementia.
3. Time processing ability and daily time management should be evaluated to inform intervention for persons with dementia.

## 1 INTRODUCTION

In most societies, the ability to manage time is of great importance for independence in daily life, participation in society and well‐being (Christiansen & Townsend, 2010; Pemberton & Cox, 2013; Topo et al., 2007). Persons with dementia often need frequent support in daily occupations that require time management skills (Boger et al., 2014; Evans & Collier, 2019; Persson et al., 2020). Reminders to keep track of appointments, to initiate, perform and finish occupations in time, as well as support in planning occupations in a specific time order might be necessary (Boger et al., 2014; Evans & Collier, 2019; Persson et al., 2020). Problems in daily time management may also lead to increased levels of stress and anxiety, for example, the person with dementia might lack the ability to estimate how long one should wait for something to happen (Nygård & Johansson, 2001). Consequently, interventions targeting occupational performance affected by impaired time processing ability should be given high priority by occupational therapists. Assessments of occupational performance and the underlying cognitive functions form the basis for interventions and, thus, access to valid and reliable assessments that can guide clinical decisions and intervention planning is essential (Fawcett, 2013; Stigen et al., 2018). The use of standardised measures can enhance clarity and consistency between professionals, whereas the clients receive improved services in which assessments and outcome data are based on valid and reliable measures (Fawcett, 2013). There is, however, a lack of instruments assessing time processing ability and daily time management in persons with dementia, which is the focus of this article.

The International Classification of Function, Disability, and Health describes time concepts in terms of both body function and activity and participation levels (World Health Organisation [WHO], 2017). The concepts defining the body functions of time are included in mental functions, where experience of time is defined as the specific mental functions of the subjective experiences related to the length and passage of time (Janeslatt, 2012; Janeslatt et al., 2009; WHO, 2017). Orientation to time comprises the mental functions that produce awareness of today, tomorrow, yesterday, clock time, and the day, month, and year (WHO, 2017). Being part of higher‐level cognitive functions, time management includes ordering events in chronological sequence and allocating different amounts of time to events and activities (Janeslätt et al., 2014; White et al., 2013; WHO, 2017). Previous research has shown that three levels in body functions—experience of time, orientation to time, and time management—can be considered as a single time processing ability construct (Janeslatt, 2012; Janeslätt et al., 2008). Although time processing ability relates to the mental (cognitive) functions required for managing time in daily life, daily time management relates to how time is actually managed in daily life (Sköld & Janeslatt, 2017). In the activity and participation domain, daily time management is part of the concept carrying out a daily routine, which includes carrying out the complex and coordinated actions needed to plan, manage, and complete day‐to‐day procedures such as budgeting time and making plans for separate activities (WHO, 2017). Daily time management includes the concept managing one's time, which is defined as managing the time required to complete usual or specific activities, such as preparing for departures from the home, taking medications, and accessing assistive technology (ICF Update Platform, 2020; Sköld & Janeslatt, 2017).

Although general instruments assessing cognitive functioning, such as Mini Mental State Examination (MMSE) and The Montreal Cognitive Assessment (MOCA), include assessments of some aspects of time orientation, they do not cover all three levels of time processing ability and daily time management (Folstein et al., 1975; Nasreddine et al., 2005; Persson et al., 2020). Available instruments such as the ICF‐based Kit for Time‐Processing Ability (KaTid®), Time‐Self‐Rating (Time‐S©), and Time‐Proxy© have been developed for other patient groups (Janeslatt et al., 2009; Janeslätt et al., 2010; Persson et al., 2017; Sköld & Janeslatt, 2017). Evaluation of the psychometric properties have shown that KaTid® measures one construct, time processing ability, whereas Time‐S© and Time‐Parent scale© measure daily time management, all in children and youth with and without disabilities. However, these instruments have not yet been tested for use with persons with dementia. It has been recommended that the psychometric properties should be evaluated when an instrument is used in a different population (Souza et al., 2017). As presented in Table 1, KaTid‐Senior is an objective test of cognitive functions, whereas Time‐S is a self‐rating and Time‐Proxy a proxy‐rating test of the ability to manage time in daily life.

**TABLE 1:** Overview of KaTid‐Senior, Time‐S Senior, and Time‐Proxy

Instrument	Type of measurement	Measures	ICF level^a^	Variable measured and ICF code^a^
KaTid‐Senior	Objective test	Cognitive functions	b body functions	Time processing ability, three levels: b1802 experience of time b1140 orientation to time b1642 time management
Time‐S Time‐P	Self‐rating Proxy‐rating	Ability to manage time in daily life	d activity and participation	Daily time management: d2305 managing ones time

^a^ International Classification of Functioning, Disability and Health (ICF).

Rasch analysis is a widely used modern test theory approach for assessing the psychometric properties when developing new instruments as well as when evaluating existing ones (Bortolotti et al., 2013). A Rasch‐based analytical approach generates validity and reliability estimates of both persons and items that are independent of the sample distribution. These estimates are used in Rasch analysis when evaluating rating scale functioning, internal scale validity, person‐response validity, person‐ and item‐separation reliability, and internal consistency (Linacre, 2002). Rasch analysis examines the internal construct validity of an instrument for unidimensionality, that is, all items have to measure a single underlying construct (Tennant & Conaghan, 2007). The main assumption in Rasch analysis is that the probability of responding correctly or incorrectly to a single item is related to both person ability and difficulty of the item (Bond & Fox, 2015). Based on the odds probabilities of responses, the Rasch analysis transforms raw item scores into interval measures, where the items are distributed in order of difficulty (from easiest to most difficult) and the individuals are distributed in order of ability (from least skilled to most skilled) (Bond & Fox, 2015; Lima et al., 2019). Such linear measures can provide meaningful guidance to both researchers and clinicians (Boone & Noltemeyer, 2017).

Access to valid and reliable instruments assessing time‐related impairments in dementia is important. Detection of time processing ability and daily time management problems at an early stage of dementia can, for example, enable timely introduction of time assistive technology and increase the possibility of a successful implementation (Persson et al., 2020). Furthermore, limited resources in health care services generates a need for time‐efficient assessments. An instrument assessing all three levels of time processing ability (KaTid‐Senior) used in combination with self‐ and proxy ratings of daily time management (Time‐S Senior and Time‐Proxy, respectively) could form a useful starting point for further investigations of the persons with dementia's individual need of support. Therefore, the aim of this study was to evaluate the psychometric properties of KaTid‐Senior, Time‐S Senior, and Time‐Proxy when used with persons with dementia.

The study addressed the following research questions:

1. What is the validity and reliability of the KaTid‐Senior when used with persons with dementia?
2. What is the validity and reliability of Time‐S Senior when used with persons with dementia?
3. What is the validity and reliability of Time‐Proxy when used with significant others of persons with dementia?

## 2 METHODS

This cross‐sectional study was approved by the Regional Ethical Review Board in Uppsala (reg.no. 2018/059). All participants gave written informed consent to participate in the study. This data collection is part of a larger project, Managing time with dementia.

### 2.1 Participants

Persons with dementia (*n* = 53) and significant others (*n* = 49) such as spouses and children were included in this cross‐sectional, non‐experimental study.

The inclusion criteria for persons with dementia were diagnosed dementia, daily time management problems identified by occupational therapists in memory assessments, age ≥60 years, ability to communicate in Swedish, and no mental illness unrelated to dementia. Exclusion criterion was a MMSE result ≤11 (Folstein et al., 1975; Kudlicka et al., 2019).

The inclusion criteria for significant others were knowledge about the person with dementia's daily life and daily time management, and the ability to communicate in Swedish.

### 2.2 Materials

#### 2.2.1 Measures

Demographic variables for persons with dementia and significant others were collected with a study‐specific questionnaire that included questions about gender, age, family situation (single, living alone; partner, not living together; partner/family, living together) and education (primary school; secondary school; college/university).

#### 2.2.2 KaTid®

KaTid© is standardised and validated for patient groups with cognitive and physical impairments and can be used to evaluate interventions (Janeslatt, 2012; Janeslatt et al., 2009; Janeslätt et al., 2010, 2014; Persson et al., 2017). The instrument evaluates the following concepts: experience of time, orientation to time, and time management (Table 1) (WHO, 2017). The version used in this study, KaTid‐Senior, was based on KaTid‐Youth which contains 59 items with good psychometric properties including a person separation of 2.11 (reliability 0.82). In a principal component analysis (PCA), variance explained by measures was 81.5%; unexpected variance in first contrast was 1.5%, providing support for validity and unidimensionality (Janeslatt, 2012). KaTid‐Senior included 29 items approximated to fit the capacity of persons with mild to moderate dementia, 25 of the items are scored Can (1), or Cannot (0), and four items are scored Can (2), On the way (1), and Cannot (0). Examples of items in each subcategory in KaTid‐Senior is provided in Appendix A.

#### 2.2.3 Time‐S Senior

Time‐S© is an instrument for self‐rating of daily time management that has been validated for adults with and without mental health conditions. The results showed principal component analysis (PCA) of 68.9% and the unexplained variance in first contrast was 3%, indicating that Time‐S measures one construct, assumed to be daily time management. The person separation was 2.75 (rel. 0.88) (Janeslätt et al., 2015). In this study, Time‐S Senior was adapted for persons with mild to moderate dementia by removing items non‐relevant for elderly persons with dementia, for example, items specific to adults who are still working, and replacing the omitted items with new relevant items. This version is called Time‐S Senior. Time‐S Senior contains 21 items with four response alternatives in a Likert scale: Never (1), Sometimes (2), Often (3), and Always (4). (Appendix B).

#### 2.2.4 Time‐Proxy

Time‐Proxy© is an instrument for proxy‐rating of a person's daily time management based on the Time‐Parent scale, which has been validated and tested for internal consistency, for children and adolescents with and without disabilities, with a Cronbach alpha of 0.79–0.86 (Janeslatt et al., 2009; Janeslätt et al., 2008). In this study, significant others' ratings were used. In Time‐Proxy non‐relevant items specific to adolescents were removed and items relevant for adults were added. Time‐Proxy contains 13 items with five response alternatives in a Likert scale: ‘I do not know’ (0), Never (1), Sometimes (2), Often (3) and Always (4). (Appendix C).

#### 2.2.5 Mini Mental State Examination

The Swedish version of the MMSE‐SR was used to measure cognitive level in persons with cognitive impairments and dementia (Folstein et al., 1975).

### 2.3 Procedures

Twelve registered occupational therapists working with memory assessments on memory clinics in 10 different regions in Sweden recruited the study participants and carried out the data collection during 2018–2021. The recruitment was conducted in connection with the memory assessments if there was an indication of daily time management problems. After the memory assessments, the occupational therapists collected the study data, at the same time or at a subsequent occasion, in clinical settings or in the participants' homes. The study data were collected by the occupational therapists using the demographic questionnaire, KaTid‐Senior form, and Time‐S Senior form for persons with dementia. Meanwhile, the significant others filled in the demographic questionnaire and the Time‐Proxy form. All occupational therapists had received training in using the instruments before the data collection started. Information on dementia diagnosis and MMSE results was obtained from medical records. If the MMSE results were done more than 6 months ago, a new MMSE assessment was conducted by the occupational therapists.

### 2.4 Statistical analyses

Rasch analyses were conducted to examine the psychometric properties of the instruments when used with persons with dementia. The Rasch partial credit model was applied to KaTid‐Senior because it allows for items with more than two response options with different scoring criteria. The Rating Scale Model was applied to Time‐S Senior and Time‐Proxy as both instruments use Likert scale types for all response options (Linacre, 2002). Psychometric properties were evaluated for each instrument.

First, the *rating scale functioning* was examined. According to Linacre's guidelines (Linacre, 2002), the average calibration for each rating category should advance monotonically and the *z*‐values in Outfit *MnSq* for each rating category should be <2. The probability curve of each rating category in the three instruments were examined separately. Every rating category was expected to be the most probable category for some part of the underlying construct. Generally, at least 10 answers for each category and item are recommended (Bond & Fox, 2015; Boone & Noltemeyer, 2017). However, this was not feasible with the sample size in this study, so a rating scale analysis of all items together using the Rating Scale model was also conducted for each instrument (Holmefur & Krumlinde‐Sundholm, 2016).

Goodness‐of‐fit statistics for *internal scale validity* and *person‐response validity* were evaluated with a focus on infit *MnSq* because infit statistics are more informative when exploring atypical response patterns on items and the fit of items to the Rasch model, whereas outfit statistics are more sensitive to outliers (Bonsaksen et al., 2013; Fan et al., 2020; Linacre, 2020). The criteria for acceptable goodness‐of‐fit for both items and persons were set at a *MnSq* ≤ 1.4 with an associated standardised *z* ≤ 2.0 (Fallahpour et al., 2014; Sköld & Janeslatt, 2017). For acceptable person‐response validity, no more than 5% of the sample can demonstrate poor goodness of fit. (Bonsaksen et al., 2013; Fan et al., 2020; Linacre, 2020).

A principal component analysis of the residuals was used to examine the *unidimensionality* of the scales, and thereby, minimise the risk of additional explanatory factors in the measures. The eigenvalue of the first contrast was set to ≤3, and at least 50% of the total variance should be explained by the first latent dimension (time processing ability and daily time management, respectively) (Fan et al., 2020; Linacre, 2020; Sköld & Janeslatt, 2017).

The *person‐separation reliability* was evaluated to distinguish between high and low performers. Low person‐separation values with a relevant person sample implies that the instrument may not be sensitive enough and that more items may be needed. The person‐separation index is an estimate of the spread or separation of persons on the measured variable. A person‐separation index of ≥1.5 is considered acceptable, ≥2 is good, and ≥3 is excellent. The person‐reliability index is the estimate of the replicability of person ordering that can be expected if a particular sample of persons were to be given another set of items measuring the same construct. The person‐reliability index should be ≥0.80 (Bond & Fox, 2015; Fan et al., 2020).

The targeting of item difficulty to person ability was inspected via person‐item maps that provide a visual illustration of both persons and items displayed along the same linear continuum. The mean values from item and person measures were also compared. Ideally, the mean values should not differ more than 0.5 logits (Linacre, 2002).

To further assess the reliability if the items measure the same construct, *the internal consistency* was evaluated for each instrument using Cronbach's alpha. A value >0.80 was considered acceptable (Bonsaksen et al., 2013; Scanlan, 2018).

After the initial Rasch analysis of the original instruments, a stepwise process was conducted whereby items failing to meet the criteria (as stated above) were removed one at a time. Person misfits were also analysed. The decision to omit persons or not was guided by weighing together information from all steps of the analysis. When omitting a person did not significantly affect the test values, or when the misfit might be explained by other reasons than random responses, no removal was done (Linacre, 2020).

Descriptive statistics for sample characterisation was calculated with SPSS v.25. The WINSTEPS analysis software, version 4.5.1, was used to conduct the Rasch analyses (Linacre, 2020).

## 3 RESULTS

### 3.1 Characteristics of participants

Almost half of the persons with dementia were women (47.2%), with a mean age of 74.9 years (Table 2). Most of the persons with dementia were living together with a partner (81.1%) and had up to secondary school education (67.9%). Alzheimer's disease was the dominant type of dementia (71.7%), and the mean result of the MMSE was 22.4 out of 30.

**TABLE 2:** Basic characteristics of participants with dementia and their significant others

Persons with dementia (*n* = 53)		Significant others (*n* = 49)	
Gender *n* (%)		Gender *n* (%)	
Women	25 (47.2)	Women	28 (57.1)
Men	28 (52.8)	Men	21 (42.9)
Age, years (mean, SD)	74.9 (6.9)	Age, years (mean, SD)	67.1 (9.7)
Family situation, *n* (%)		Relation to participant, *n* (%)	
Single, living alone	7 (13.2)	Spouse	41 (83.7)
Partner, not living together	3 (5.7)	Child	7 (14.3)
Partner/family, living together	43 (81.1)	Missing information	1 (2.0)
Education *n (%)*		Support given (days/week), *n* (%)	
Primary school	22 (41.5)	≤1	6 (12.2)
Secondary school	14 (26.4)	2–3	6 (12.2)
College/university	16 (30.2)	Daily	35 (71.4)
Missing information	1 (1.9)	Missing information	2 (4.1)
Diagnosis, *n* (%)			
Alzheimers disease	38 (71.7)		
Vascular dementia	6 (11.3)		
Dementia with Lewy bodies	4 (7.5)		
Dementia (other)	3 (5.7)		
Alcohol dementia	1 (1.9)		
MMSE (mean, SD)	22.4 (4.0)		

More than half of the significant others were women (57.1%), with a mean age of 67 years. Most of them were a spouse of the persons with dementia (83.7%) and gave daily support to the persons with dementia (71.4%).

Time‐Proxy data was available from 49 out of 53 participants.

### 3.2 Psychometric properties for KaTid‐Senior

A brief summary of the process and key results is provided in Table 3, drawing upon examples by Bonsaksen and colleagues (2013).

**TABLE 3:** Overview of Rasch analyses of KaTid‐Senior, Time‐S Senior and Time‐Proxy

Psychometric property	Statistical approach and criteria	KaTid‐S original, 29 items, 53 PwD^a^	KaTid‐S, 26 items, 52 PwD^a^	KaTid‐S final version 26 items, 53 PwD^a^	Time‐S original 21 items 53 PwD^a^	Time‐S 17 items, 50 PwD^a^	Time‐S final version 17 items, 53 PwDs	Time‐P original 13 items, 49 SO^b^	Time‐P final version 10 items, 49 SO^b^
**Rating scale functioning** Does the rating scale function consistently across items?	Average measures for each step category should advance monotonically *z*‐values <2.0 in outfit MnSq values for step category calibrations	Rating scale met criterion except for two items Rating scale met criterion except for three items	Rating scale met criterion Rating scale met criterion	Rating scale met criterion Rating scale met criterion except for two items	Rating scale met criterion except for two items Rating scale met criterion except for two items	Rating scale met criterion Rating scale met criterion	Rating scale met criterion Rating scale met criterion except for two items	Rating scale met criterion except for two items Rating scale met criterion except for two items	Rating scale met criterion Rating scale met criterion except for one item
**Internal scale validity** Do the item responses match the expected responses? Is the scale unidimensional?	Item goodness‐of‐fit statistics *Infit MnSq* values ≤1.4 and *Zstd* ≤2.0 Principal component analysis. Eigenvalue of the first contrast ≤3 ≥50% of total variance explained by first component	One item failed to meet criterion 3.24 61.6	All items met criterion 2.81 55.1	All items met criterion 2.72 55.1	Two items failed to meet criterion 3.29 53.9	All items met criterion 2.57 52.1	All items met criterion 2.79 53.3	Two items failed to meet criterion 2.36 53.9	All items met criterion 2.27 52.1
**Person‐response validity** Do the individual responses match expected responses?	Person goodness‐of‐fit statistics.*Infit MnSq* values ≤1.4 and *Zstd* ≤2.0≤5% fails to demonstrate acceptable goodness‐of‐fit values	1 person (1.9%) failed to meet criterion	All persons met criterion	1 person (1.9%) failed to meet criterion	4 persons (7.5%) failed to meet criterion	All persons met criterion	4 persons (7.5%) failed to meet criterion	2 persons (4.1%) failed to meet criterion	1 person (2.0%) failed to meet criterion
**Person‐separation reliability** Can the scale distinguish persons into different levels?	Person separation index ≥1.5 is acceptable, ≥2 is good, ≥3 is excellent. Person reliability index ≥0.80.	1.60 0.72	1.82 0.77	1.82 0.77	2.86 0.89	2.83 0.89	2.70 0.88	2.46 0.86	2.93 0.90
**Internal consistency** Are item responses equivalent with each other? (reliability)	Cronbach's alpha coefficient >0.8	0.77	0.81	0.81	0.92	0.93	0.92	0.92	0.93

^a^ Persons with dementia (PwD).

^b^ Significant others (SO).

**FIGURE 1:**
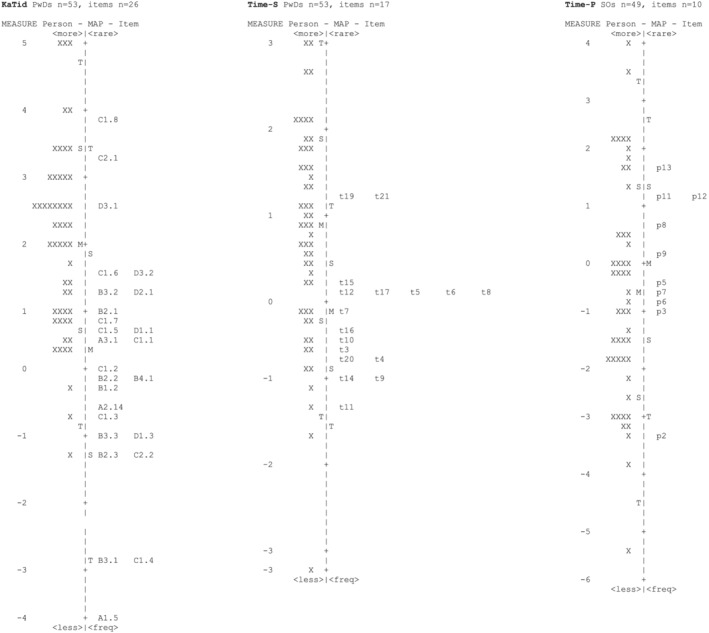
Person‐item maps for KaTid‐Senior, Time‐S Senior and Time‐Proxy

In the first step, the functioning of the rating scale was examined. In the original version of KaTid‐Senior, the average measures for each step category advanced monotonically for all items except two, and the *z*‐values in Outfit *MnSq* for each rating category were <2 for all items except three. After a stepwise removal of three items (A3.2 *Reproduce 10 seconds*, D1.2 *How long does it take to unload the dishwasher into the cupboards*, and D4.1 *For how long do you think we have been doing this?*) and one person with misfit, all items met the criterion for the rating scale. However, analysis of the one‐person misfit revealed a random response pattern where the individual failed to respond correctly on one of the easiest items but responded correctly on the most difficult items despite a low total score. Thus, the overall assessment was that the rating scale in KaTid‐Senior works for this target group even if two items were disordered due to the misfitting person's divergent response pattern. When evaluating the internal scale validity, infit statistics were sound for all remaining items in the final version. According to the principal component analysis of the final version, the first component explained ≥50% of total variance and the Eigenvalue of the first contrast was ≤3, which indicates unidimensionality of time processing ability. Regarding the person‐response validity, all three analyses of KaTid‐Senior presented in Table 3 demonstrated acceptable goodness‐of‐fit to the Rasch model. The person‐separation index for the final version of KaTid‐Senior was acceptable (1.82), but the person‐separation reliability was low (0.77). The Cronbach's alpha coefficient was acceptable (0.81). The person‐item map (Figure 1) showed that the person ability was higher than the item difficulty, which was also supported by the mean difference of 1.67 logits. These results indicate a need to add more difficult items to match the ability of this target group.

### 3.3 Psychometric properties for Time‐S Senior

When evaluating the rating scale functioning in the original version of Time‐S Senior, the average measures for each step category advanced monotonically and the *z*‐values in Outfit *MnSq* for each rating category were <2 for all items but two. After removing four items and three persons, the rating scale functioning met the set criteria. Removal of four items with misfit (t1 *I sleep well at night*, t2 *It is my responsibility to wake up and get ready in the mornings*, t13 *I keep track of when it is time to do the laundry*, and t18 *I myself keep track of what is going to happen next week*) were also required for Time‐S Senior to meet the criterion for internal scale validity. The principal component analysis value for unidimensionality, that is the measurement of daily time management, was not acceptable for the original version of Time‐S Senior, but for the version with four items and three persons omitted and for the final version with four items omitted the values were within limits for the set criteria. For both versions, the principal component analysis also showed that the first component was explained by ≥50% of total variance and that the Eigenvalue of the first contrast was ≤3. In the original version of Time‐S Senior, four persons did not meet the criteria for person‐response validity (7.5%), but after a stepwise omission of three persons the criteria were met. However, further analyses showed that one person had an overall differing response pattern with low variance, where 14 of the 29 items were scored ‘Never’, and four items were given high scores of ‘Most times’ or ‘Always’. Three participants (one female and two male) had two misfitting item responses each and all of them were scored lower than expected (‘Never’), maybe because these items were not relevant to the individuals and there was a need for other response alternatives. Therefore, in the final version of Time‐S Senior no persons were omitted, resulting in misfit of four persons and 7.5% failure to meet criterion. The person‐separation index and person‐reliability index for Time‐S Senior were good in all versions, indicating separation of individuals into different levels of daily time management. The Cronbach's alpha coefficient was also good (0.92 in the final version). However, the person‐item map showed that the person ability was higher than the item difficulty (Figure 1) and the mean difference in the final version was 0.89 logits.

### 3.4 Psychometric properties for Time‐Proxy

In the original version of Time‐Proxy, two items did not meet the criterion for rating scale functioning. When the response alternative ‘Do not know’ was treated as a missing value, all items met criterion for average measures. One item touched the limit for outfit *MnSq z*‐values < 2.0, the others met the criterion. Regarding the internal scale validity, the removal of three items (p1 *Sleeps well at night*, p4 *Can wait without getting angry or troubled*, and p10 *Has routines for getting to bed on time*) resulted in sound infit statistics for the remaining items. The principal component analysis showed acceptable values in both the original and final version of Time‐Proxy, indicating unidimensionality and the measurement of one construct, daily time management. All versions of Time‐Proxy demonstrated acceptable values for person‐response validity, person‐separation reliability, and internal consistency. The person‐item map for the final version of Time‐Proxy (Figure 1) showed that the person ability was lower than the item difficulty. The mean difference was −.51 logits.

## 4 DISCUSSION

The aim of this study was to evaluate the psychometric properties of KaTid, Time‐S Senior, and Time‐Proxy when used with persons with dementia.

The results showed that the revised version of *KaTid‐Senior* is a valid measure with potential to be a useful clinical instrument by assessing all three levels of time processing ability for persons with dementia. Such an instrument has not previously been developed for this patient group (Persson et al., 2020). KaTid‐Senior demonstrated acceptable psychometric values, indicating measurement of a single construct, namely time processing ability (Bond & Fox, 2015; Linacre, 2020). However, the targeting was low. There is a need, therefore, to add more challenging items to increase the accuracy and to better capture time processing ability impairments in persons with dementia with very mild symptoms. At the same time, keeping the items that were too easy for this sample could allow for measurement of change over time and might better target persons with dementia with more severe dementia symptoms. Moreover, the rating scale functioning must be given further attention. Although the overall interpretation is that the rating scales in KaTid‐Senior is functioning as intended, further evaluations with larger samples is required to confirm the validity (Van Zile‐Tamsen, 2017).

The final version of *Time‐S Senior* demonstrated good internal scale validity, person‐response and person‐separation reliability, and an indication of unidimensionality (daily time management). However, the rating scale functioning including individual responses and step category calibrations was not sufficient. Additional analyses of the misfitting persons indicated, for example, that answers that did not fit into the model were related to items that might be gender‐specific and not related to the person's ability level. Person‐response validity issues need to be further investigated, in particular when it comes to self‐ratings among persons with dementia as limited awareness of impairments is a common consequence of dementia. (Snow et al., 2005; Wadley et al., 2003). Furthermore, decreased episodic memory functions in dementia can affect the ability to remember specific situations and relate them to questions when rating daily time management in Time‐S Senior. This might explain the high ratings of person ability in relation to item difficulties. On the other hand, research has established the relevance and importance of persons with dementias' experiences and self‐ratings (Martyr & Clare, 2018; Öhman et al., 2008). A previous interview study found that persons with Alzheimer's disease were able to express awareness of the consequences of dementia through reflections on everyday occupations (Öhman et al., 2008). By detecting self‐rated problems in daily time management, clinical interventions can address areas of daily time management that may be highly relevant to the person with dementia. Still, for this purpose Time‐S Senior needs to be further developed to fit this target group. In Time‐S Senior, the person with dementia scores the frequency of a set of predetermined activities without the possibility to specify whether the activity is relevant, or how important the performance is. Research has also shown that persons with dementia often need support from their significant others in activities that require daily time management (Persson et al., 2020). Thus, it could be a problem to respond adequately in the current version of Time‐S Senior. One way of improving the validity of the psychometric properties of Time‐S Senior could be to clarify and specify the questions and scoring. Moreover, a non‐applicable response option should be added. Additional response options might enhance the understanding of the persons with dementias' daily time management.

Generally, the psychometric properties of the revised version of *Time‐Proxy* were satisfactory. One exception was the rating scale step category calibrations where one item touched the limit of *z*‐value <2.0 in outfit *MnSq*. However, the reduction of 13 items to 10 might entail a need to add other items. Comparable items that work well in Time‐S Senior might be useful and, in turn, facilitate comparisons between the person with dementia's self‐ratings and proxy ratings. Proxy‐ratings are widely used in research and in clinical settings, but there are important aspects to consider. Unlike KaTid‐Senior and Time‐S Senior, the person‐item map for Time‐Proxy displayed lower person ability than item difficulty. This is in line with previous research showing that proxy ratings, in comparison with self‐ratings in situations where both can be achieved, often tend to be more negative (Crespo et al., 2013; Martyr & Clare, 2018). Thus, it is important to establish the accuracy of ratings of daily time management made by both persons with dementia and their significant others in relation to objective assessments of time processing ability, and further development of the instruments is needed.

### 4.1 Limitations of the study and implications for future research

A limitation of this study was the relatively small sample size. During periods, COVID‐19 restrictions significantly reduced the possibility for the memory clinics to recruit new participants. However, one of the advantages with Rasch analysis is that the technique can be used also with small samples to validate measures (Linacre, 2020). Both infit and outfit *MnSq* statistics are relatively insensitive to sample size variation (Smith et al., 2008). Moreover, the few missing data in this study were considered random and could thus be handled by the Rasch analyses (Bond & Fox, 2015).

Further development and evaluation of adjusted versions of KaTid‐Senior, Time‐S Senior, and Time‐Proxy for persons with dementia are important for future research and clinical use. More demanding items should be added to KaTid‐Senior to better target persons with dementia with mild symptoms because this could enhance the possibility of early detection of time processing ability impairments and thus the possibility to provide early targeted interventions. Also, the items and rating scales in Time‐S Senior should be further adjusted for persons with dementia because cognitive impairments can make self‐ratings more difficult. Confirming valid rating scale functioning in all three instruments is another important issue and the rating scales need to be examined based on more observations. Additional psychometric properties such as interrater and test–retest reliability has not yet been investigated for KaTid‐Senior, Time‐S Senior, and Time‐Proxy when used with persons with dementia.

Observational instruments such as KaTid‐Senior can add more in‐depth knowledge about persons with dementias' time processing ability and self‐ratings, and proxy‐ratings of daily time management are important complements for a client‐centred approach, where Time‐S Senior and Time‐Proxy can support health care professionals in identifying and evaluating their clients' own perceptions of daily time management. The relationship between self‐ratings and proxy ratings of daily time management, measures of time processing ability, and severity of dementia should be explored to increase the knowledge about time‐related assessments of persons with dementia.

## 5 CONCLUSION

KaTid‐Senior, Time‐S Senior, and Time‐Proxy have been evaluated for persons with dementia for the first time, showing acceptable psychometric properties. The Rasch analysis indicated unidimensionality of time processing ability or daily time management, respectively. However, some issues should be addressed to improve the measures. Addition of more difficult items in KaTid‐Senior could increase the accuracy and thus better target persons with mild dementia. The response alternatives in Time‐S Senior need to be adjusted and evaluated to better fit persons with dementia and to increase the person‐response validity, and Time‐Proxy could be further developed to facilitate comparisons with Time‐S Senior. Nevertheless, the findings have important implications for the possibility to assess time processing ability and daily time management impairments validly and reliably in persons with dementia in clinical research and healthcare settings. In turn, this can contribute to the development of targeted methods to compensate for impaired time processing ability and daily time management in persons with dementia, for example, prescription of a time assistive technology that matches the level of time processing ability. The results indicate that the assessments could enable early detection of impairments in time processing ability and problems in daily time management, thereby facilitating adequate timing of interventions and enhancing occupational performance.

## CONFLICTS OF INTERESTS

The last author originally developed the instruments KaTid, Time‐S, and Time‐Proxy and sells KaTid material and training courses to professionals. The other authors report no conflicts of interest.

## AUTHOR CONTRIBUTIONS

Development of research questions; design of analytic strategy; drafting, critically revising and approving manuscript: All authors. Data analyses and interpretation of results: AP and GJ.

## Data Availability

The data that support the findings of this study are available from the corresponding author upon reasonable request.

## APPENDIX A ITEMS IN KATID®‐SENIOR, EXAMPLES IN EACH SUBCATEGORY

### A. TIME PERCEPTION – TIME SENSE

A1.5 Match a minute – an hour (pictures)

A2.14 Match a minute – quarter of an hour (pictures)

A3.1 Reproduce 10 seconds (stopwatch by the persons left ear)

A3.2 Reproduce 10 seconds (stopwatch by the persons right ear)

### B. TIME ORIENTATION/TIME CONCEPTS

B1.2 Sorting pictures in a time sequence

B2.1 What day is it today?

B3.1 Which month is Christmas?

### C. TIME ORIENTATION, OBJECTIVE TIME

C1.2 Which of these times is the same as on the clock? (picture)

C1.6 Time between 13:50 and 14:30?

C2.2 Set a timer on 8 minutes

### D. TIME MANAGEMENT

D1.2 How long does it take to unload the dishwasher into the cupboards?

D2.1 Time needed to get to the doctor on time?

D4.1 For how long do you think we have been doing this?

## APPENDIX B ITEMS AND RESPONSE ALTERNATIVES IN THE TIME‐S© SELF‐RATING SCALE

**Table:**

How often do you do it?		Never	Sometimes	Often	Always
1.	I sleep well at night				
2.	It is my responsibility to wake up and get ready in the mornings				
3.	I start tasks on my own				
4.	I complete tasks on my own				
5.	I keep track of when I have an appointment with the doctor				
6.	I manage to do the household work, for example, cleaning				
7.	In given time, I am aware of the tasks that can be done, for example, before dinner				
8.	I can cook a meal so it is ready in time				
9.	I come on time to social events, for example, parties				
10.	If I have promised to call someone, I keep in mind when to call the person				
11.	If I have agreed to meet a friend/relative, I am on time for the meeting				
12.	I keep track of the time for the TV programs I want to watch				
13.	I keep track of when it is time to do the laundry				
14.	I have a routine so that I go to bed on time				
15.	I myself plan what I want to do				
16.	I feel when it is time to finish what I am doing				
17.	I know how long different activities take, so I know what I have time to do				
18.	I myself keep track of what is going to happen next week				
19.	I myself keep track of what is going to happen this month				
20.	I myself make sure to be ready in time when something important is going to happen, for example, a meeting/doctors appointment				
21.	I take responsibility for planning events that must be planned over time, for example, planning a trip				

## APPENDIX C ITEMS AND RESPONSE ALTERNATIVES IN TIME‐PROXY©

**Table:**

My proxy:		Never	Sometimes	Often	Always	Dont know
1.	Sleeps well at night					
2.	Takes responsibility to wake up, get up and get ready in the mornings					
3.	Can start to do a task independently					
4.	Can wait without getting angry or troubled					
5.	Keeps track of when there is an appointment with the doctor					
6.	Has a good routine for doing the household chores					
7.	Can keep track of the time for having lunch					
8.	Can cook a meal so it is ready in time					
9.	Can independently be on time to social events/parties					
10.	Has routines for getting to bed on time					
11.	Keeps in mind what is happening in the coming month					
12.	Takes responsibility for tasks that need to be planned over time					
13.	Can plan a trip and be on time for an event/function					
